# Composite scoring system and optimal tumor budding cut-off number for estimating lymph node metastasis in submucosal colorectal cancer

**DOI:** 10.1186/s12885-022-09957-8

**Published:** 2022-08-06

**Authors:** Jeong-ki Kim, Ye-Young Rhee, Jeong Mo Bae, Jung Ho Kim, Seong-Joon Koh, Hyun Jung Lee, Jong Pil Im, Min Jung Kim, Seung-Bum Ryoo, Seung-Yong Jeong, Kyu Joo Park, Ji Won Park, Gyeong Hoon Kang

**Affiliations:** 1 Department of Surgery, Chung-Ang University Hospital, 06973 Seoul, Republic of Korea; 2 Chung-Ang University College of Medicine, 06973 Seoul, Republic of Korea; 3 Pathology Center, Seegene Medical Foundation, 05542 Seoul, Republic of Korea; 4 Department of Pathology, Seoul National University College of Medicine, 03080 Seoul, Republic of Korea; 5 Department of Internal Medicine, Seoul National University College of Medicine, 03080 Seoul, Republic of Korea; 6 Department of Surgery, Seoul National University College of Medicine, 03080 Seoul, Republic of Korea; 7 Cancer Research Institute, Seoul National University, 03080 Seoul, Republic of Korea

**Keywords:** Colorectal neoplasm, Histopathology, Lymph nodes metastasis, Tumor budding

## Abstract

**Background:**

Tumor budding is associated with lymph node (LN) metastasis in submucosal colorectal cancer (CRC). However, the rate of LN metastasis associated with the number of tumor buds is unknown. Here, we determined the optimal tumor budding cut-off number and developed a composite scoring system (CSS) for estimating LN metastasis of submucosal CRC.

**Methods:**

In total, 395 patients with histologically confirmed T1N0–2M0 CRC were evaluated. The clinicopathological characteristics were subjected to univariate and multivariate analyses. The Akaike information criterion (AIC) values of the multivariate models were evaluated to identify the optimal cut-off number. A CSS for LN metastasis was developed using independent risk factors.

**Results:**

The prevalence of LN metastasis was 13.2%. Histological differentiation, lymphatic or venous invasion, and tumor budding were associated with LN metastasis in univariate analyses. In multivariate models adjusted for histological differentiation and lymphatic or venous invasion, the AIC value was lowest for five tumor buds. Unfavorable differentiation (odds ratio [OR], 8.16; 95% confidence interval [CI], 1.80–36.89), lymphatic or venous invasion (OR, 5.91; 95% CI, 2.91–11.97), and five or more tumor buds (OR, 3.01; 95% CI, 1.21–7.69) were independent risk factors. In a CSS using these three risk factors, the rates of LN metastasis were 5.6%, 15.5%, 31.0%, and 52.4% for total composite scores of 0, 1, 2, and ≥ 3, respectively.

**Conclusions:**

For the estimation of LN metastasis in submucosal CRC, the optimal tumor budding cut-off number was five. Our CSS can be utilized to estimate LN metastasis.

**Supplementary Information:**

The online version contains supplementary material available at 10.1186/s12885-022-09957-8.

## Introduction

Colorectal cancer (CRC) is a major cause of cancer-associated mortality and is the most common cancer worldwide [1]. CRC can be cured by surgical treatment if detected early (stage I) without additional chemotherapy [2]. Early CRC is increasingly detected by CRC screening. In early CRC cases, malignant polyps without deep invasion can be treated via endoscopic resection. Further radical surgery may be needed according to the probability of lymph node (LN) metastasis. The identification of risk factors for LN metastasis can assist in formulating a treatment strategy.

The prevalence of LN metastasis in submucosal CRC is 0–17.3% [3]. The risk factors for LN metastasis in submucosal CRC include histopathological features, such as lymphatic or venous invasion [4–7], poorly differentiated carcinoma [8, 9], deep submucosal invasion [9–11], and tumor budding [4–6, 12]. In addition, tumor volume, morphological features, mode of growth, absence of background adenoma, and/or lymphoid infiltration are histopathological factors associated with LN metastasis [8, 13, 14]. Tumor budding is defined as isolated single cells or clusters of up to four cells at the invasive margin [15]. Tumor budding is an adverse factor in CRC [16–20]. Furthermore, tumor budding is a predictive parameter for LN metastasis according to the guidelines of the European Society for Medical Oncology [21] and the Japanese Society for Cancer of the Colon and Rectum [22]. To our knowledge, few studies have evaluated the optimal point of tumor budding for estimating LN metastasis in submucosal CRC. Here, we determined the optimal tumor budding cut-off number and developed a scoring system to estimate LN metastasis of submucosal CRC.

## Materials and methods

### Study design and ethics

This was retrospective study determined the optimal tumor budding cut-off number and developed a scoring system to estimate LN metastasis of submucosal CRC. This study was reviewed and approved by the Institutional Review Board of the Seoul National University Hospital Biomedical Research Institute (approval number: H-2107–045-1232). All patients provided written informed consent and approval were obtained from all patients. All procedures were carried out in accordance with the relevant guidelines and regulations.

### Patients

In total, 12,749 patients underwent surgery for CRC at Seoul National University Hospital from January 1, 2002, to December 31, 2019. Among them, patients who underwent a radical operation for submucosal CRC and had available histopathological reports were eligible for this study. Submucosal CRC was defined as an adenocarcinoma that invaded the submucosal layer and conformed to the classification guidelines of the American Joint Committee on Cancer Staging. Patients who received neoadjuvant therapy or underwent local resection were excluded. Finally, 395 patients were enrolled.

### Data collection and pathological review

Data concerning patients’ clinicopathological parameters were collected from the electronic medical records. Sex, age, body mass index, American Society of Anesthesiologists score, carcinoembryonic antigen level, and tumor location were analyzed as clinical characteristics. Right colon cancer was defined as tumors in the cecum, ascending colon, and transverse colon; left colon cancer was defined as cancers in the descending and sigmoid colon; and rectal cancer was defined as tumors in the recto-sigmoid junction and the rectum [23].

**Fig. 1:**
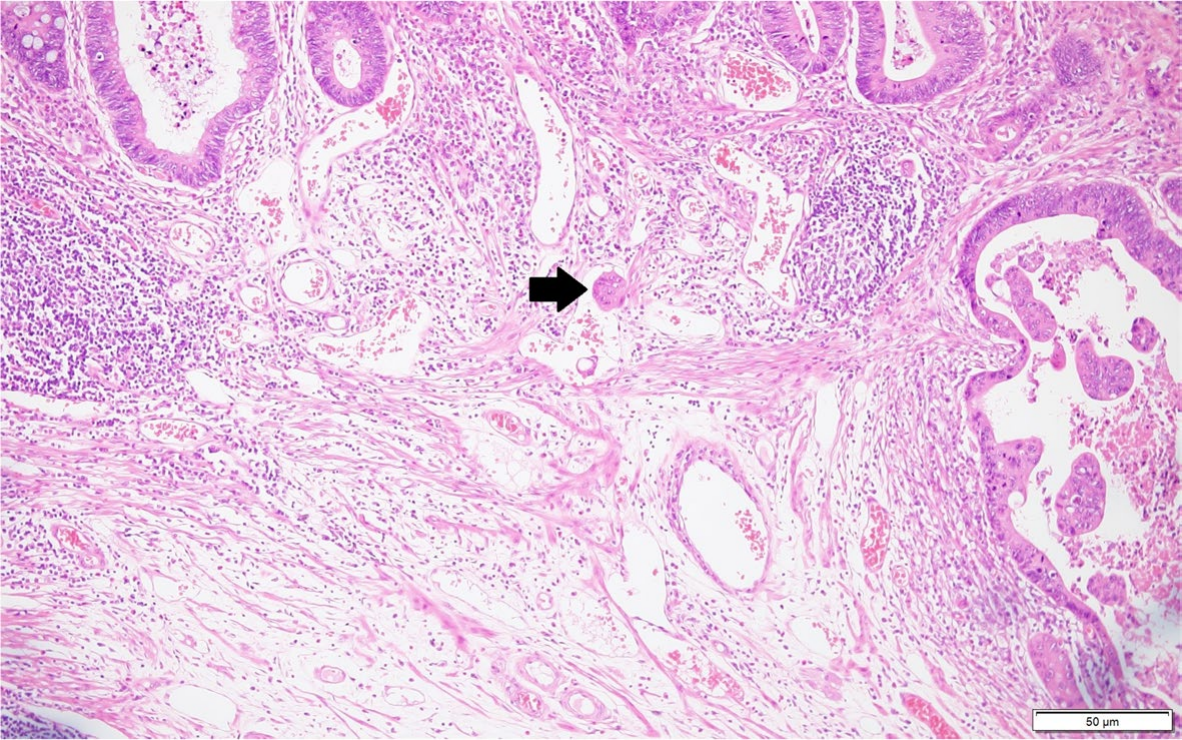
Representative histopathological image of lymphatic or venous invasion (hematoxylin and eosin staining, × 200)

**Fig. 2:**
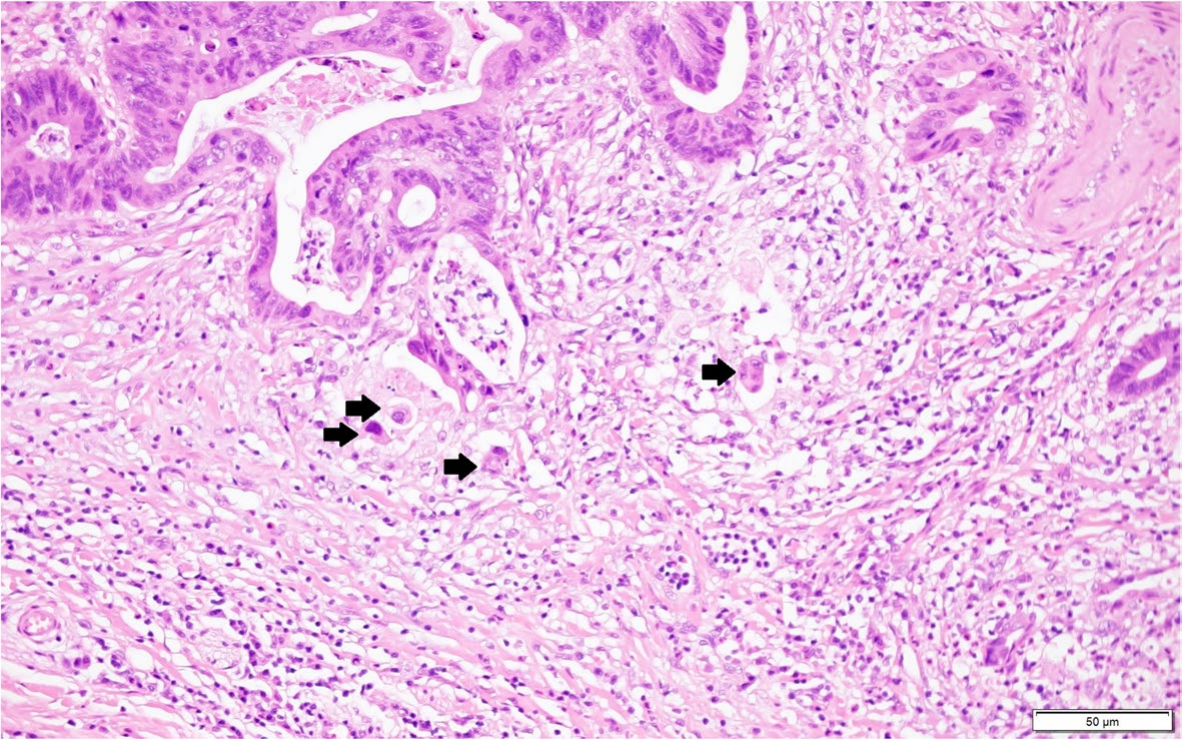
Representative histopathological image of tumor budding, isolated single cells, or clusters of up to four cells (hematoxylin and eosin staining, × 200)

Pathological features assessed were tumor histological type, lymphatic or venous invasion, perineural invasion, number of tumor buds, distance from the proximal to the distal margin, number of harvested LNs, and number of metastasized LNs. Tumors were histologically classified as favorable differentiation (well or moderately differentiated carcinoma) or unfavorable differentiation (poorly differentiated, undifferentiated, signet ring cell, or mucinous carcinoma), in accordance with the World Health Organization guidelines. Pathological features were evaluated via hematoxylin and eosin staining alone. Lymphatic or venous invasion was considered present when tumor cells invaded non-muscle-walled small vessels or large vessels with a smooth muscle layer and/or an elastic lamina layer (Fig. 1) [24]. Perineural invasion was considered present when tumor cells reached the peripheral nerve sheath layers. To objectively evaluate tumor budding, we confirmed the existence of isolated single cells or clusters of up to four cells via hematoxylin and eosin staining of tumor tissues; the number of tumor budding in a microscopic field was verified at × 200 magnification (Fig. 2). Pathological slides were assessed by three experienced gastrointestinal pathologists. The assessment of tumor budding was performed by another pathologist.

### Statistical analysis

The clinical characteristics and pathological features were compared according to LN metastasis status to identify risk factors for LN metastasis. Pearson’s χ^2^ test or Fisher’s exact test was used to compare categorical variables; Student’s *t*-test was used to compare continuous variables. Multivariate logistic regression analysis was performed to identify independent risk factors that were predictive of LN metastasis. We used the Akaike information criterion (AIC) of the adjusted multivariate models to identify the optimal tumor budding cut-off number. The tumor budding cut-off values, from 0 to 12 at intervals of 1, were assessed as an indicator of LN metastasis based on the AIC. The best model exhibited the lowest AIC value. The cut-off value was determined when the model had the lowest AIC value. A composite scoring system (CSS) was developed to estimate LN metastasis by adding rounded values of the coefficients of independent risk factors. All statistical analyses were performed using SPSS 22 software (IBM Corp., Armonk, NY, USA). A *p*-value < 0.05 was considered statistically significant.

## Results

The prevalence of LN metastasis was 13.2% (52/395). No clinical characteristics significantly differed between patients with and without LN metastasis (Table 1). Correlations between LN metastasis and pathological features are shown in Table 1. Tumors with LN metastasis had a higher prevalence of unfavorable differentiation (*p* = 0.022) and lymphatic or venous invasion (*p* < 0.001). The number of buds was higher in tumors with LN metastasis than in tumors without LN metastasis (*p* = 0.001).

**Table 1:** Univariate analysis of Lymph node (LN) metastasis

Parameter	LN (-), *n* = 343	LN ( +), *n* = 52	*P*
Age^a^	63 (23–87)	64 (32–84)	0.570^c^
Sex			
Male	225 (65.6%)	30 (57.7%)	0.340
Female	118 (34.4%)	22 (42.3%)	
BMI ^b^			
Mean	23.9 ± 2.9	23.7 ± 2.95	0.628^d^
ASA grade			
I	107 (31.2%)	15 (28.8%)	0.957
II	218 (63.6%)	34 (65.5%)	
III	14 (4.1%)	2 (3.8%)	
unknown	4 (1.1%)	1 (1.9%)	
CEA^a^	1.5 (0.4–39.8)	1.6 (0.5–18.2)	0.380
Location			
Right colon	80 (23.3%)	10 (19.2%)	0.320
Left colon	153 (44.6%)	21 (40.4%)	
Rectum	109 (31.8%)	20 (38.5%)	
unknown	1 (0.3%)	1 (1.9%)	
Histological type			
Favorable	321 (93.6%)	46 (88.5%)	0.022
Unfavorable	5 (1.5%)	4 (7.7%)	
unknown	17 (4.9%)	2(3.8%)	
Lymphatic or venous invasion			
Negative	304 (88.6%)	28 (53.8%)	< 0.001
Positive	36 (10.5%)	21 (40.4%)	
unknown	3 (0.9%)	3 (5.8%)	
Perineural invasion			
Negative	324 (94.5%)	48 (92.3%)	1.000
Positive	2 (0.5%)	0 (0%)	
unknown	17 (5.0%)	4 (7.7%)	
Tumor Budding			
Low budding (0–4 buds)	285 (83.1%)	31 (59.6%)	< 0.001
Intermediate budding (5–9 buds)	33 (9.6%)	11 (21.2%)	
High budding (10 or more buds)	25(7.3%)	10 (19.2%)	
Tumor budding (n)^a^	0 (0–32)	3 (0–15)	< 0.001^c^
Tumor size (cm)^a^	1.6 (0.2–10.5)	1.65 (0.1–19.0)	0.912^c^
Harvest LNs (n)^a^	16 (0–165)	16 (2–46)	0.647^c^
Proximal margin (cm)^a^	11.0 (1.4–136.0)	10.0 (3.5–89.0)	0.486^c^
Distal margin (cm)^a^	3.5 (0–75.0)	4.75 (0.5–48.0)	0.094^c^

*Abbreviations: BMI* Body mass index, *ASA* American Society of Anesthesiologists, *CEA* Carcinoembryonic antigen

^a^ Median (range);

^b^ Mean ± standard error of the mean (SEM);

^c^ Mann–Whitney U test;

^d^ Student’s t-test

**Fig. 3:**
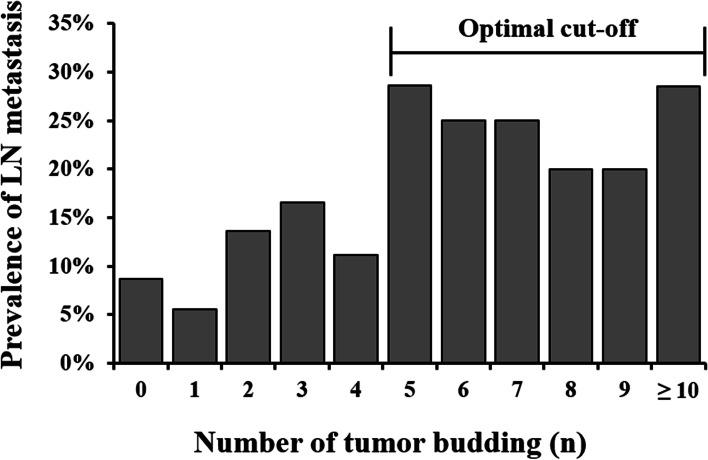
The prevalence of lymph node (LN) metastasis is associated with the number of tumor budding

**Fig. 4:**
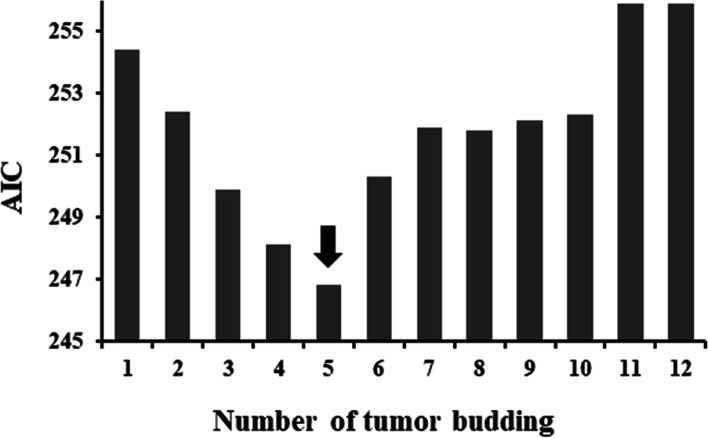
Akaike information criterion (AIC) in logistic regression models adjusted for histological type and lymphatic or venous invasion

Figure 3 shows the prevalence of LN metastasis and the tumor budding value. The prevalence of LN metastasis in tumors with five tumor buds was 28.6%. Tumors with five or more buds had higher rates of LN metastasis than did tumors with fewer than five tumor buds. The cut-off number was determined using the AIC from the tumor budding logistic regression models, adjusted for histological type and lymphatic or venous invasion. The AIC value was lowest (246.8) for tumors with five buds (Fig. 4). Thus, the optimal tumor budding cut-off number was five.

**Fig. 5:**
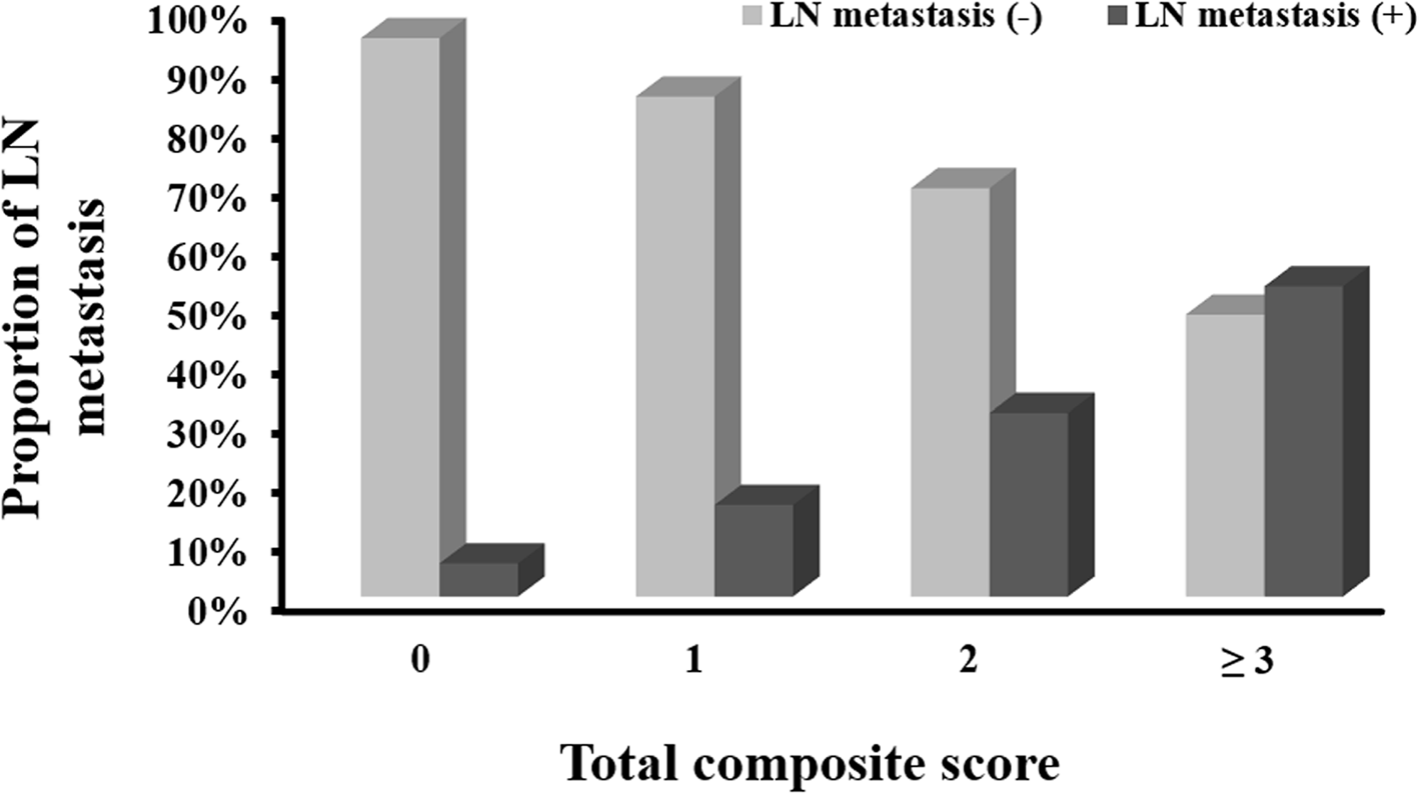
Proportion of lymph node (LN) metastasis according to the total composite score. Total composite score: 2 × histological type [favorable differentiation, 0; unfavorable differentiation, 1] + 2 × lymphatic or venous invasion [negative, 0; positive, 1] + 1 × tumor budding [< 5/HPF, 0; ≥ 5/HPF, 1]

In a multivariate analysis using a cut-off of five, unfavorable differentiation (odds ratio [OR], 8.16; 95% confidence interval [CI], 1.80–36.89; *p* = 0.006), positive lymphatic or venous invasion (OR, 5.91; 95% CI, 2.91–11.97; *p* < 0.001), and tumor budding (≥ 5/high-power field [HPF]; OR, 3.01; 95% CI, 1.21–7.69; *p* = 0.002) were significant predictive parameters for LN metastasis (Table 2). In the multivariate model, the coefficients for histological type, lymphatic or venous invasion, and tumor budding were 2.10, 1.78, and 1.10, respectively. By adding rounded coefficients, a composite score was developed (2 × histological type [favorable differentiation, 0; unfavorable differentiation, 1] + 2 × lymphatic or venous invasion [negative, 0; positive, 1] + 1 × tumor budding [< 5/HPF, 0; ≥ 5/HPF, 1]). Higher composite scores were associated with higher rates of LN metastasis (5.6%, 15.5%, 31.0%, and 52.4% for total composite scores of 0, 1, 2, and ≥ 3, respectively; Fig. 5).

**Table 2:** Multivariate analysis of Lymph node (LN) metastasis

Parameter	OR	95% CI	*P*
**Histological type** (unfavorable *vs*. favorable)	8.16	1.80–36.89	0.006
**Lymphatic or venous invasion** (positive *vs*. negative)	5.91	2.91–11.97	< 0.001
**Tumor budding** (≥ 5 *vs*. < 5/HPF)	3.01	1.21–7.69	0.002

## Discussion

For the estimation of LN metastasis in submucosal CRC, the optimal tumor budding cut-off number was five. Imai reported that tumor budding or sprouting reflects faster tumor growth [25]. In 1989, tumor budding was defined by Morodomi as a collection of isolated cancer cells without a distinct structure (i.e., undifferentiated). Because the cells appeared to bud out from a large cancer gland, they were termed “budding” [26]. In addition, to determine the degree of budding, a pathological tissue slide was divided into four areas with dimensions of 500 × 2,500 µm, and the mean number of buds per area was calculated [25]. The Japanese classification defines a tumor budding as an isolated single cell or cluster of cells consisting of fewer than five cells at the invasive margin of a tumor [27]. The International Tumor Budding Consensus Conference (ITBCC) established an evidence-based, standardized scoring system for CRC tumor budding, which was defined as a single cell or a cluster of ≤ 4 tumor cells. Tumor budding was graded using a three-tier system; scores of 0–4 indicate low budding (Bd1), 5–9 indicate intermediate budding (Bd2), and ≥ 10 indicate high budding (Bd3). Tumor budding is an independent predictor of LN metastasis in patients with pT1 colorectal cancer. Tumor budding is assessed in a hotspot (a field of 0.785 mm^2^) of the invasive front [15]. In the present study, buds were enumerated using a 10 × ocular lens at 20 × magnification, in accordance with the Japanese classification. The tumor budding grade according to the number of buds in a 0.785-mm^2^ field was defined as follows: grade 1, 0–4; grade 2, 5–9; and grade 3, ≥ 10 [27]. A multicenter study by the Budding Investigation Project Committee of the Japanese Society for Cancer of the Colon and Rectum, in which grade 1 was defined as low grade and grade 2/3 was defined as high grade, showed that a high grade was associated with LN metastasis [27]. When LN metastasis status was verified according to the number of tumor budding, the OR for five or more buds was 8.0 [26]. In the present study, the rate of LN metastasis increased as the number of tumor buds increased. The AIC value was lowest with five tumor buds; thus, five was the optimal cut-off value, consistent with the definition of low grade in the Japanese classification. The presence of five or more buds was independently associated with LN metastasis. The LN metastasis rate associated with high budding (≥ 10 buds; Bd3) was comparable to the LN metastasis rate associated with intermediate budding (5–9 buds; Bd2) (Tables S1 and S2). The impact of Bd3 status on LN metastasis was similar to the impact of Bd2 status on LN metastasis. Thus, a cut-off value of 5 is reasonable.

The histopathological predictors of LN metastasis in submucosal CRC were the depth of invasion (submucosal invasion ≥ 1,000 µm), unfavorable differentiation (poorly differentiated, mucinous carcinoma, or signet-ring cell carcinoma), and lymphatic or venous invasion. We confirmed that unfavorable differentiation and lymphatic or venous invasion were independent predictors in a multivariate analysis. Ryu et al. reported that lymphatic invasion and histopathological differentiation were significant risk factors in 179 patients with early CRC [28]. In a meta-analysis, lymphatic invasion was the most important predictor of LN metastasis; histological grade was also a key predictor [29]. The European Society for Medical Oncology, Japanese Society for Cancer of the Colon and Rectum, National Comprehensive Cancer Network, and Korean clinical practice guidelines recommend additional radical operations after endoscopic resection of submucosal cancer in patients with an unfavorable histological type, deep submucosal invasion, lymphatic or venous invasion, or tumor budding [21, 22, 30, 31]. We did not employ immunohistochemical staining when evaluating lymphatic or venous invasion. Although staining of the endothelial or elastic tissues of venous walls by D2-40 (Podopalnin) or Elastica van Gieson may increase the accuracy of evaluation, these methods are not routinely employed because of the high costs and laboratory challenges imposed [32]. A limitation of the present study was that it did not confirm the depth of invasion. However, a population-based cohort study demonstrated that age < 60 years, mucinous carcinoma, lymphovascular invasion, and perineural invasion were independent predictive factors, whereas deep submucosal invasion was not significant in the multivariate analysis (*p* = 0.075), for patients with submucosal CRC undergoing a radical operation [33].

The prevalence of LN metastasis is 10–15% in patients who undergo additional operations after endoscopic resection [33–35]. In our study, the prevalence of LN metastasis was 13.2%. Most patients without LN metastasis are at risk of surgical complications. To avoid unnecessary radical surgery and failure to identify LN metastasis, a more precise predictive model for LN metastasis is needed. Several prediction models for LN metastasis in submucosal CRC have been developed [36–38]. The least absolute shrinkage and selection operator prediction model includes histopathological factors [36]; nomograms that included independent clinicopathological factors have also been used to estimate LN metastasis [37, 38]. These predictive models have good discriminatory power. We developed a simple prediction scoring system for LN metastasis that can be applied in daily clinical practice. The relative risk of LN metastasis increased as the total composite score increased. Patients with a total composite score ≥ 2 had a LN metastasis rate > 30%. Additional surgery is recommended for these patients.

To our knowledge, this is one of few studies to investigate the optimal tumor budding cut-off number. Our work had some limitations. First, the study was retrospective in nature; selection bias may have been present. Second, the study was performed in a single institution and enrolled a small number of patients. Moreover, we did not evaluate the invasion depth when deriving risk factors. A prospective, multi-center study is needed to obtain more accurate and detailed results.

## Conclusions

For the estimation of LN metastasis in submucosal CRC, the optimal tumor budding cut-off number was five. Our CSS can be utilized to estimate LN metastasis.

## Supplementary Information

**Additional file 1:** **Table S1**. Lymph node (LN) metastasis status according to the three-tier system of tumor budding. **Table S2.** Multivariate analysis of lymph node (LN) metastasis status according to the three-tier system of tumor budding.

## Data Availability

The datasets generated and/or analyzed during the current study are not publicly available due to ethical restrictions but are available from the corresponding author on reasonable request.

## Abbreviations

AIC: Akaike information criterion
ASA: American Society of Anesthesiologists
BMI: Body mass index
CEA: Carcinoembryonic antigen
CI: Confidence interval
CRC: Colorectal cancer
CSS: Composite scoring system
HPF: High-power field
ITBCC: International Tumor Budding Consensus Conference
LN: Lymph node
OR: Odds ratio
