# Supplementary material for: Composite scoring system and optimal tumor budding cut-off number for estimating lymph node metastasis in submucosal colorectal cancer
Source: BMC Cancer. 2022 Aug 6;22:861. doi: 10.1186/s12885-022-09957-8 (PMC9357306; doi:10.1186/s12885-022-09957-8)
Supplement: Supplementary file 1 — Additional file 1: Table S1. Lymph node (LN) metastasis status according to the three-tier system of tumor budding. Table S2. Multivariate analysis of lymph node (LN) metastasis status according to the three-tier system of tumor budding. [file 12885_2022_9957_MOESM1_ESM.docx]

**Table S1**: Lymph node (LN) metastasis status according to the three-tier system of tumor budding

| **Parameter** |  | **LN (-), n = 343** | **LN (+), n = 52** | ***P-*value** |
| --- | --- | --- | --- | --- |
| Tumor budding | Low budding, Bd1  (0–4 buds) | 285 (90.2%) | 31 (9.8%) | < 0.001 |
|  | Intermediate budding, Bd2  (5–9 buds) | 33 (75.0%) | 11 (25.0%) |  |
|  | High budding, Bd3  (≥ 10 buds) | 25 (71.4%) | 10 (28.6%) |  |

**Table S2**: Multivariate analysis of lymph node (LN) metastasis status according to the three-tier system of tumor budding

| **Parameter** |  | **OR** | **95% CI** | ***P*** |
| --- | --- | --- | --- | --- |
| **Histological type** | Favorable | Ref |  |  |
|  | Unfavorable | 8.16 | 1.80–36.89 | 0.006 |
| **Lymphatic or venous invasion** | Negative | Ref |  |  |
|  | Positive | 5.91 | 2.91–11.97 | < 0.001 |
| **Tumor budding** | Low budding, Bd1  (0–4 buds) | Ref. |  |  |
|  | Intermediate budding, Bd2  (5–9 buds) | 2.98 | 1.25–7.12 | 0.014 |
|  | High budding, Bd3  (≥ 10 buds) | 3.06 | 1.21–7.69 | 0.018 |
